# The Report of Dr. Forbes Winslow to the Right Hon. The Lords Justices of the Court of Appeal

**Published:** 1852-04-01

**Authors:** 


"To the Right Hon. the Lord Chancellor."

---

### THE REPORT OF DR. FORBES WINSLOW TO THE RIGHT HON. THE LORDS JUSTICES OF THE COURT OF APPEAL.

"My Lords,—In obedience to the instructions contained in your lordships' order, I have the honour to report, for your lordships' information, that I have professionally visited Mrs. Catherine Cumming at her residence, No. 59, Queen's-road, St. John's-wood, and have subjected her to several examinations, for the purpose of ascertaining the condition of her mind, and competency to manage herself and her affairs. I had two interviews with her, of some duration, previously to her removal from the lunatic asylum at Brixton; I have also visited her since her discharge from the asylum and return to her own residence, on the 22nd, 23rd, 24th, 25th, 26th, 27th, and 28th of November last, and the 1st and 2nd of December instant.

"I have also had a lengthened conversation with Mr. Ince, the son-in-law of Mrs. Cumming, and have perused several affidavits and medical certificates made by parties on both sides in this matter, with the view of acquainting myself with the facts of the case, and the grounds upon which Mrs. Cumming is alleged to be insane.

"I have also had a conversation, upon two different occasions, with Mr. Turner, the solicitor acting for the children of Mrs. Cumming in support of the application for a commission of lunacy.

"The grounds upon which Mrs. Cumming is alleged to be insane are the following:—

"1st. The manifestation on the part of Mrs. Cumming of an unnatural and insane antipathy to her children.

"2nd. Her representation that some years back her daughter, Mrs. Ince, had endeavoured to strangle her.

"3rd. Her assertion that an attempt was made a few years ago to poison her, by means of oxalic acid.

"The above seem to be the principal, and I think the only evidence, apart from the alleged existence of a general incapacity to manage her property referred to in the medical certificates and affidavits, with the view of establishing Mrs. Cumming's insanity. Upon these three points I have examined Mrs. Cumming with considerable care, and at great length. I found, upon my referring to her two daughters, Mrs. Ince and Mrs. Hooper, and to her two sons-in-law, Mrs. Cumming became somewhat, but by no means violently, excited. She admitted that she had been for years at variance with them, but that her antipathy entirely arose from the cruel treatment to which she alleged her children had subjected her. Upon asking for an explanation, she replied, that various efforts had been made by her two daughters and sons-in-law to obtain possession of her property, and to deprive her of personal liberty; that she had been confined for some months in an asylum; that her daughters had, without good or valid reason, instituted proceedings against her, with the view of depriving her of the control of her property, and confining her for life as a lunatic; that at their suggestion, and upon their petition, a commission of lunacy was granted by the Lord Chancellor, but that the matter had been compromised by her children, on condition that she (Mrs. Cumming) would make some assignment of her property to them. Mrs. Cumming complained of having been systematically persecuted for a number of years, and stated that this had engendered in her mind the feeling of dislike which had been adduced as a symptom of her mental derangement. She declared that previously to this ill-treatment she loved her children affectionately.

"Upon asking if she had other reasons for her somewhat unnatural feelings, she said that Mrs. Ince had greatly offended her by encouraging, contrary to her known wishes and protest, a clandestine marriage between her eldest daughter, Thomasine, and a person very much inferior to her in position of life, whose name was Hooper, and who acted as trumpeter to a band. Mrs. Cumming observed, 'That it was natural that she should object to a union so opposed to her daughter's interests and station in life, and that if she had not objected to the match, she would have been fit for a lunatic asylum.' She then again stated, that if her daughters had not made so many efforts to turn her out of her own house, and to obtain possession of her property, she would not have felt towards them any antipathy. 'How can I like my children,' Mrs. Cumming feelingly observed, 'when I recollect how they have treated their mother? But they wanted my money. If I had been poor, they would have left me alone.'

"I then examined Mrs. Cumming with reference to the second allegation—viz., the declaration that her daughter, Mrs. Ince, had attempted to strangle her. Mrs. Cumming's explanation of the matter is in substance as follows:—

'She alleges, that pending the proceedings which the family had instituted against her, with the view of establishing her insanity, her mind was kept in a constant state of alarm and excitement from an apprehension that an attempt was in contemplation to carry her to a lunatic asylum. Knowing, also, that her two daughters were the acting agents in the matter, and had presented a petition to the Court of Chancery, with the view of depriving her of the management of her property, she felt indisposed to see them. That it was during the time whilst her mind was thus absorbed with the idea of what she conceived to be the harsh, cruel, and unnatural conduct of her children, that Mrs. Ince, without giving any intimation of a visit, rushed into her sitting-room, and violently flung her arms round her neck. That she was so surprised by this unexpected burst of apparent affection, and bearing in mind the proceedings Mrs. Ince had commenced against her, she exclaimed, 'Have you come to strangle me?' or, 'You wish to strangle me.' She maintains it was natural for her, under the circumstances, to have an apprehension of the kind, knowing that her children were plotting against her liberty.

"I then referred to the third fact, adduced as proof of Mrs. Cumming's insanity—viz., that poison had been infused into her milk.

"Mrs. Cumming asserts that her milk was poisoned; she will not say by whom, or for what purpose; but she persists in the declaration that such was the fact, and that the said milk was analyzed by a physician, to whom it was sent, and that he subjected it to proper tests, and found poison in it.

"Considering the first allegation of Mrs. Cumming's insanity—viz., her antipathy to her family, which is alleged to be morbid, and the result of delusive impressions, I would observe, that it is a common feature of disordered minds for such impressions to take possession of the imagination, without there existing the slightest justification for the feeling. A distinction, however, ought to be made between an antipathy which is clearly the effect of a delusion, and a morbid perversion of the natural instincts, and that dislike and aversion which are traceable to, and the probable result of, a course of conduct pursued by those against whom the antipathy is directed. The natural operation of the feelings must not be confounded with those deviations from the healthy current of thoughts the obvious and undoubted products of a mind in an insane condition.

"The matter in dispute in regard to the first allegation resolves itself into a question of fact. Was the conduct of Mrs. Cumming's children such as at all to justify the mother in entertaining so strong, and apparently so unnatural a feeling towards them? If it can be established that they have invariably treated Mrs. Cumming with the kindness, respect, gentleness, and deference due from children to a parent; if it can be proved that she is under a delusion in regard to the daughters having made several attempts to deprive their mother of her personal liberty, and to dispossess her of the control of her property; that no proceedings upon the petition of the children had been instituted to place Mrs. Cummjng under the jurisdiction of the Court of Chancery; then I should feel disposed to believe the feeling of aversion manifested by Mrs. Cumming to be morbid in its character, and that her mind is in an unsound state.

"What are, however, the facts of the case? It appears that during her late husband's life, Mrs. Cumming was confined as a lunatic, and in that proceeding her children took an active part, Captain Cumming, her husband, being at the time bedridden. Without wishing for one moment to question the humanity and necessity of the steps taken by Mrs. Cumming's family, on that or any other occasion when Mrs. Cumming's personal free agency had been interfered with, it is important, for a right elucidation of the question, to look at the facts of the case. The first confinement in the asylum took place in May, 1846. In August, 1846, after the death of Captain Cumming, the daughter, or daughters, presented a petition for a commission of lunacy. A commission was issued. Pending this investigation, Mrs. Cumming was restored to her own residence. The inquiry into Mrs. Catherine Cumming's insanity took place in the month of September, 1846, and extended over a period of ten days, during which time Mrs. Cumming was in court, and was subjected by the commissioner to a long examination. The question of Mrs. Cumming's lunacy never went to the jury, as a compromise was proposed and agreed to. The nature of the compromise was as follows:—That the petitioners were to withdraw all further proceedings; that Mrs. Cumming should be immediately discharged from all restraint; that three trustees should be appointed, in whose names her property was to be invested, one to be named by Mrs. Ince and Mrs. Hooper, and one by Mrs. Cumming, and one by the Commissioner. A deed of settlement was to be prepared, under which Mrs. Cumming was to be entitled to the rents and profits of her estates for her life; after her death, one-third of the annual income was to be held by trustees for the separate use of Mrs. Ince for her life, after her death for her husband; and Mrs. Cumming was to have power of appointment over the remaining third; and if power not exercised, then such remaining third to be divided between Mrs. Ince and Mrs. Hooper, &c., &c. The compromise in question seems to have been signed on the 29th of April, 1847, by two gentlemen of eminence at the bar.

"On the 27th of November, 1851, the family again adopted active measures to deprive Mrs. Cumming of her liberty; and on the joint certificates of Drs. King, of Brighton, and Sir A. Morison, M.D., of London, (the order being signed by Mrs. Ince,) Mrs. Cumming was forcibly taken from her own apartments at Brighton, and brought up to London, and placed in an establishment for lunatics at Brixton.

"It would appear from the previous statement, that Mrs. Cumming's family have, on different occasions (influenced, it may be, by the most humane, but, perhaps, mistaken motives), endeavoured to guard Mrs. Cumming against the operations of extraneous influences by throwing about her person and property the protection of the law. It is not for me to sit in judgment upon their conduct, or to attribute any but the most proper motives for the course they thought necessary to adopt. That the children have been obliged to put themselves prominently forward in the proceedings is an undoubted fact, and one with which Mrs. Cumming appears to be well acquainted. Looking at the case with a knowledge of these circumstances, it becomes a legitimate question whether the antipathy mentioned by Mrs. Cumming has not been engendered in her mind by the course which her children have considered it their duty to take? Whether it is not a natural and not a diseased antipathy? I cannot bring my mind to the conclusion that the aversion which Mrs. Cumming manifests towards her children is the result either of delusive impressions, or the consequence of the perverted affections of a disordered mind. It is possible, considering Mrs. Cumming's advanced age, her natural violence and irritability, her great bodily suffering, and the excitement to which her mind had been exposed for so many years, that she may attach undue importance to facts, and be disposed to be more suspicious of the conduct of others than a person of different temperament, age, and in the vigour of life, would exhibit under similar circumstances. The fact of a compromise having been made during the late Commission of Inquiry, and all proceedings having been abandoned upon the condition of Mrs. Cumming assigning her property to trustees, affords to Mrs. Cumming a reasonable pretext for the impression that her family are not anxious about her person, provided they could obtain possession of her property. I do not say such is a legitimate or logical inference, but is it not a natural one? I therefore dismiss the first ground of insanity, as not, in my judgment, established; and proceed to consider the second fact referred to—viz., the assertion of Mrs. Cumming that her daughter, Mrs. Ince, endeavoured to strangle her.

"When questioned upon this subject, Mrs. Cumming observes, that whatever idea she might formerly have entertained upon the matter, she does not now believe that Mrs. Ince had any such intention as that which she in a moment of irritation and fright attributed to her. She states, that at the time when she used the language from which an inference was drawn that she charged Mrs. Ince with an attempt to strangle her, she was greatly incensed by what she thought to be the harsh and unnatural proceedings of her children. Her belief was, that they would leave no stone unturned to obtain possession of her person and property. With the view of satisfying my mind as to whether Mrs. Cumming was purposely concealing the alleged delusion, I questioned her very closely, but could detect no evidence of the fact. Admitting that such an exclamation was made, I question whether, in the right signification of the term, we should be justified in the opinion that it was a delusion. Ought it not to be viewed (looking at the circumstances by which she was then surrounded) more in the light of an erroneous conclusion—a wrong deduction, than as the creation of a disordered imagination? I therefore dismiss the second allegation of insanity as inconclusive.

"The gravest, and certainly the most important, symptom in Mrs. Cumming's case, I have now to consider—viz., her belief that oxalic acid had been infused into some milk, with the view of poisoning her. As Mrs. Cumming pertinaciously adheres to her assertion that poison was, after analysis, detected in the milk, I have thought it my duty to see and examine the physician to whom the milk was sent for analysis. I am assured by that gentleman, Dr. Robert Barnes, of Gloucester-terrace, Hyde-park, a physician of character and respectability, that he detected poison in the milk, in the form of the superacetate of lead. Of this fact Dr. Barnes says he is prepared to swear. I think the mistake made by Mrs. Cumming as to the nature of the poison is not entitled to any weight in the consideration of the question at issue. The third allegation of insanity ought, after this explanation, to be at once dismissed, as Mrs.

Cumming's impression with regard to the poison appears to have been a fact, and not a delusion. The question suggests itself, how was Mrs. Cumming aware of there being poison in the milk prior to its being subjected to analysis? She informed me that her suspicions were excited by the fact of her cat having refused to drink the milk, and in consequence of several of her fowls having been found dead in the garden; they having been killed, as I am told, by the orders of Mr. Haynes.

"With the view of satisfying myself as to the existence of any impairment of the intellect, incapacitating Mrs. Cumming for the care of herself and the management of her property, apart altogether from the delusions with which she is represented to be afflicted, I subjected her to a careful examination. Mrs. Cumming is capable of conversing rationally and continuously upon most topics, without exhibiting more defect of memory or impairment of the understanding than we have a right to expect in a lady seventy-five years of age, and with her amount of physical suffering. At present it is difficult to abstract Mrs. Cumming's mind from the contemplation of her own distressing case. She informed me that her income ranged between 400*l.* and 500*l.* a-year, and was derivable from property situated in Monmouthshire. I examined her particularly as to the manner in which she had disposed of her property, and what papers she had signed. Her replies to these questions were not as satisfactory as I could desire. She said she had unbounded confidence in her solicitor, Mr. Haynes, who had for many years managed her private affairs; and who had acted most kindly and honourably towards her, and who had, from time to time, rendered to her an account of his proceedings. I should not infer that Mrs. Cumming, at any period of her life, had devoted much attention to her own affairs, from, perhaps, a natural indisposition to attend to matters of business.

"Mrs. Cumming admitted that she had been living beyond her income, but that her reason for so doing was, that as her children had behaved so unkindly to her, she felt little disposition to save money for their advantage. I questioned Mrs. Cumming particularly as to the future disposition of her property. She assured me that if her liberty was guaranteed, she had no desire to disinherit her children; but if they persisted in dragging her from her own house into an asylnm, at her advanced age, and with her acute bodily suffering, she saw no reason why her children should possess one penny of her property after her death. Having been informed by Mr. Turner, the solicitor acting for the family, in support of the commission, that prior to Captain Cumming's death and Mrs. Cumming's first confinement as a lunatic, she had manifested some delusions in reference to her husband having had improper intercourse with the servants in the house, and that at this moment her lunacy was obvious when this matter was made the subject of conversation, I made a point of referring to it in my interviews with her. When I asked her the question, whether she had been happy during the period of her husband's life, she replied that she had no wish to revert to the subject of her husband or his faults—that he was dead and buried, and that it was her wish to forget all his faults and failings. When pressed upon the point, Mrs. Cumming observed that Captain Cumming was not exactly what she could have wished; that he certainly was guilty of irregularities, but that she had no wish to talk upon the subject. I inferred from what remarks were subsequently made by Mrs. Cumming, that she believed that her late husband had been improperly connected with one of his servants. I do not, however, think that this ought to be considered as legitimate evidence of Mrs. Cumming's present insanity. If the fact were not as she represents, being naturally suspicious and jealous, she may have given a false colouring to his conduct. Her assertion of his infidelity—her firm belief in the fact—would not, without a minute knowledge of family circumstances, at all justify me in the conclusion that Mrs. Cumming's mind is now unsound upon this point.

"I think it my duty to state that Mrs. Cumming is partially paralysed in both legs, and that she is suffering from great debility and a painful and incurable disease of the bladder, and that her general health is so much impaired, that I question whether her life will be prolonged through the present winter.

"Viewing the case of Mrs. Cumming in all its features, I have the honour to report to the Lords Justices, that I am of opinion that Mrs. Cumming is a person of sound mind, and capable of managing herself and her property.

"FORBES B. WINSLOW, M.D.

"45, Albemarle-street, December 5, 1851."

